# Is caffeine intake a risk factor leading to infertility? A protocol of an epidemiological systematic review of controlled clinical studies

**DOI:** 10.1186/s13643-016-0221-9

**Published:** 2016-03-15

**Authors:** Huijuan Cao, Jun Ren, Xue Feng, Guoyan Yang, Jianping Liu

**Affiliations:** Centre for Evidence-Based Chinese Medicine, Beijing University of Chinese Medicine, Beijing, China; World Federation of Chinese Medicine Societies, Beijing, China; Centre for Complementary Medicine Research, University of Western Sydney, Sydney, Australia

**Keywords:** Caffeine intake, Infertility, Systematic review

## Abstract

**Background:**

Previous studies showed that high dose of caffeine intake may induce some specific human reproductive system diseases, even lead to infertility.

**Objectives:**

In consideration of the high consumption of caffeine according to the latest population-based survey, this review is aimed to systematically review the evidence from all controlled clinical studies of caffeine intake for infertility.

**Designs:**

Relevant randomized/quasi-randomized controlled trials, non-randomized clinical studies, cohort studies, and case-control studies will be included in this review. Participants will be either those without a history of infertility who are willing to have a baby (for prospective studies) or infertile patients with confirmed diagnosis (for retrospective studies). Caffeine or caffeine-containing beverage will be observed as the exposure factor. The key outcome will be the diagnosis of infertility in participants. All relevant published/unpublished or ongoing studies will be searched from seven databases and four online systems until December 2015. Two authors will screen the literatures and extract the data independently. Methodological quality of the included studies will be assessed by two authors according to either Risk of Bias Assessment or Newcastle-Ottawa Scale. We will use R software to analyze the data. Dose of caffeine will be quantified on a daily basis, and relative risk with their 95 % confidence interval will be measured. If data permit, meta-analysis and dose-response analysis will be conducted. Summary of findings tables will be generated using Guideline Development Tool online.

**Systematic review registration:**

PROSPERO CRD42015015714

**Electronic supplementary material:**

The online version of this article (doi:10.1186/s13643-016-0221-9) contains supplementary material, which is available to authorized users.

## Background

Infertility is a disease of the reproductive system defined by the failure to achieve a clinical pregnancy after 12 months or more of regular unprotected sexual intercourse without a certain reason, such as breast feeding or postpartum menorrhea [1]. Primary infertility is defined as the absence of a live birth for women who desire a child and have been in a union for at least 5 years, during which they have not used any contraceptives [2]. The increase of age leads to a growth of infertility rate [3]. Infertility may be the result of infection in the man or woman, but often, there is no obvious underlying cause [1]. There are many biological factors and other reasons which may lead to infertility, including some that can be treated by medical interventions [4].

Without a certain effective treatment, in vitro fertilization (IVF)—which may result in approximately 30 % live birth—seems to be the first option for couples who suffered from infertility [5]. However, high rates of drop-out are frequently encountered in IVF treatment due to the financial burden, which is the commonest cause (65 %) [6]. Furthermore, the latest evidence showed that the consequences of infertility have greater impact on a woman’s life and can be a lifetime crisis [7].

Caffeine is present in many drinks and foods consumed during pregnancy and most notably in tea, coffee, colas, energy drinks, and chocolate [8]. Caffeine can have both positive and negative health effects. It may confer a modest protective effect against some diseases of the cardiovascular system and of the metabolism of carbohydrates and lipids, including the various forms of arterial cardiovascular disease, arrhythmia, heart insufficiency, diabetes, liver disease [9], and even Parkinson’s disease [10].

For the negative effect of caffeine intake on reproductive system diseases, we only found a systematic review [8] with observational studies that showed high dose caffeine intake may induce specific adverse events during pregnancy (abortion, low birth weight, stillbirth, et al.). Though no systematic review is concerned with the relationship between caffeine intake and infertility, many primary studies have demonstrated that caffeine is associated with infertility [11–14]. The effects of caffeine consumption on delayed conception were evaluated in a European multicenter study on risk factors of infertility, which issued that women in the highest level of caffeine consumption had an increase of 11 % in the time leading to the first pregnancy. Another retrospective study also found that nonsmokers who consumed more than 301 mg caffeine daily may have 2.65 more times chance on delayed conception. Hence, one prospective cohort study with 18,555 participants found that the intake of caffeinated soft drinks (compared the highest to lowest categories) was positively related to ovulatory disorder infertility (risk ratio = 1.47, 95 % confidence interval from 1.09 to 1.98).

Other epidemiologic literatures reported the effect of lifestyle factors, which point out the potential relationship between caffeine consumption and female infertility [15–17]. Among them, one study focused on 104 healthy women who had been attempting to become pregnant for 3 months were interviewed about their use of caffeinated beverages, alcohol, and cigarettes. Daily consumption of coffee was likely to be relevant to the pregnancy rate with interviewed women [18].

Regarding the potential mechanism of the caffeine as a risk factor for the reproductive system, one experimental study [19] examined whether in utero and lactational exposure to caffeine affects the reproductive function of the offspring of rats and found significant (caffeine) dose-related decreases in the body and reproductive organ weight, seminiferous tubule diameter, and germinal epithelium height of the offspring. The damage on testicles of the experimental animals was also demonstrated [20, 21]. Even the hormone level [22] and semen quality [23] would be affected by caffeine exposure which also induces adverse outcomes. In consideration of the high caffeine intake rate in women, even in pregnant women, it is essential to determine the relationship between caffeine intake and women infertility. Due to the absence of systematic evidence of this area, we planned to conduct this review with an evidence-based medicine approach to investigate whether caffeine intake is a risk factor for infertility.

## Objectives

This study aims to systematically review the evidence from any type of controlled clinical studies of caffeine intake for human infertility.

## Methods

Protocol of this review was registered at PROSPERO international register of systematic reviews (No. CRD42015015714) on 25 December 2014, which was available from http://www.crd.york.ac.uk/PROSPERO/display_record.asp?ID=CRD42015015714. However, regarding the purpose of this review, which is to investigate the relationship between caffeine intake and infertility, we made some revisions of the protocol and broadened the target population and type of studies. Details of the protocol are described below, and all the amendments were pre-defined before conducting the review.

### Inclusion criteria of studies

No restriction on publication types or language. For studies which reported in languages other than Chinese and English, we will ask professional interpreters to do the document translation.

#### Type of studies

We will include controlled clinical studies, in terms of randomized controlled trials (RCTs), quasi-RCTs or non-randomized clinical studies (both prospective and retrospective), cohort studies, and case-control studies.

#### Type of participants

For prospective studies, participants will be women/men without a history of infertility who are willing to have a baby. For retrospective studies, infertile women/men with confirmed diagnosis will be included. All the included women should be in their childbearing age, which means they need to be premenopausal.

#### Type of exposure factors

As an exposure factor, caffeine can be derived from coffee, tea, cola, or other caffeine-containing beverage. Though the caffeine is also present in many foods (such as chocolate), it may only provide 2 % of the caffeine consumed according to the data from two population-based surveys [24, 25]. Since the contribution of the caffeine-containing food as a source of caffeine is small, and the dose of the caffeine is difficult to count, we will only focus on studies in which caffeine or caffeine-containing beverage was observed as the exposure factor.

#### Type of outcomes

Diagnosis of infertility or not for in participants will be the key outcome of this review. Generally accepted diagnostic criteria should be mentioned in the original studies; according to which, those who were diagnosed as infertile should have suffered from at least 12 months of unsuccessful conception.

### Search strategy

We will search PubMed, the Cochrane CENTRAL Database, EMBASE, China National Knowledge Infrastructure (CNKI), VIP Database, and Chinese Biomedical Database (CBM) from inception to December 2015. Unpublished literatures (such as conference report, dissertation, etc.) will be achieved through Wanfang Database and CADTH Grey matters checklist (https://www.cadth.ca/resources/finding-evidence/grey-matters). Ongoing studies will also be searched through the metaRegister of Controlled Trials (http://www.controlled-trials.com), the US National Institutes of Health Ongoing Trials Register (www.clinicaltrials.gov), and the Australian New Zealand Clinical Trials Registry (www.anzctr.org.au). All references of the studies included will be hand-searched for additional relevant reports.

We will use abstract terms, keywords, and Medical Subject Headings (MeSH) as searching terms, including “infertility” and “sterility” with “coffee”, “caffeinated” and “caffeine”. Variations of caffeine, such as “coffein”, “calcium caffeine”, “caffeine calcium complex”, “anhydrous caffeine”, “cafeine”, “animine”, and “caffein” will also be included during the literature searching.

### Study selection

**Fig. 1:**
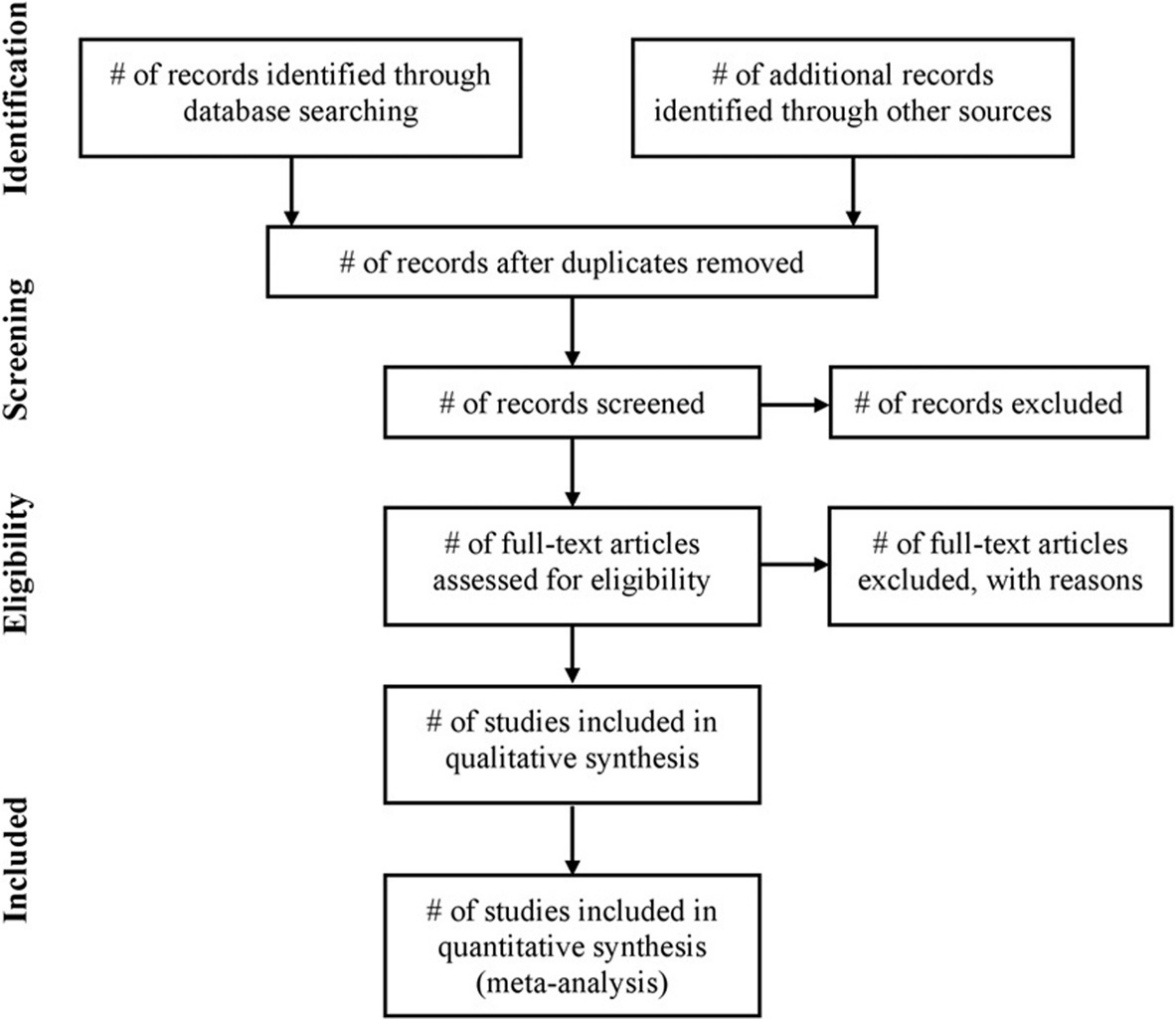
Study flow chart

NoteExpress software will be used to manage records from searching databases and do screening. Two authors (JR and XF) will independently screen and select the eligible literatures for both title/abstract and full-text phases of the review; disagreements will be resolved by discussion and, if needed, arbitrated by a third author (HJC). A pre-designed screening form will be used for to double check the eligibility of potential included trials during this process. And the form will be pilot tested as a sample of studies. If information to make a judgment regarding inclusion is not provided, we will contact the authors of the original studies. Furthermore, the study selection process will be reported in a flow diagram as shown in Fig. 1 (study flow chart).

### Data extraction

Two authors (JR and XF) will independently extract data on patient characteristics, exposure details, clinical outcomes, and quality-related information. Details of data extraction for included studies are shown in Additional files\ 1, 2, and 3 (data extraction form). The discrepancies will be resolved through consensus. Missing data will be achieved through contacting the authors of the original studies.

### Methodological quality assessment

Two authors (HJC and JR) will independently assess the methodological quality of included trials. Methodological quality of analytic studies will be assessed according to the Newcastle-Ottawa Scale (NOS) [26]. Stars were awarded in the “representative” selection samples, “comparability” between groups, and “completeness” and “validity” records of caffeine intake or infertility. The methodological quality of RCTs, quasi-RCTs, or non-randomized clinical studies will be assessed according to the risk of bias tool described in the Cochrane Handbook for Systematic Reviews of Interventions [27]. Seven elements will be assessed: random sequence generation, allocation concealment, blinding of included participants, blinding of outcome assessors, incomplete outcome data, selective reporting, and other biases. All eligible studies will be included, regardless of perceived quality.

### Data analysis

We will use R software to analyze the data. If data permit, dose of caffeine will be quantified on a daily basis. Where daily consumption of the caffeine were not presented as milligrams, a serving of coffee was assumed to contain approximately 100 mg caffeine and any other caffeine-containing beverage (e.g., tea or cola) was assumed to contain 60 mg of caffeine on average [5]. Then, dose of the caffeine is categorized according to the daily consumption, mean or median will be used for presenting caffeine intake for each category. For each single study, relative risk (including odds ratio, risk ratio, and hazard ratio) with their 95 % confidence interval will be measured for participants who have caffeine consumption compared with those who have not. Clinical heterogeneity is determined by the consistence of characteristics of participants, exposure factors (such as dose of caffeine intake), and the outcome between studies. Statistical heterogeneity among studies will be tested by *I*^2^ statistic. The 95 % confidence interval of *I*^2^ will also be calculated and reported [28]. Tau-square and its 95 % confidence interval will also be reported to further detect the between-study variance [29]. Meta-analysis will be done if there are acceptable clinical and statistical heterogeneity (*I*^2^ < 75 %) among trials. Random effects models which are more conservative and provide better estimates with wider confidence intervals will be used when conducting meta-analysis [30]. To avoid the potential confounding bias, only data adjusted for the identical pre-specified confounders will be pooled in the meta-analysis. When the detected statistical heterogeneity is significant (*I*^2^ > 75 %) among the relevant included studies, we will check the potential sources of the heterogeneity and try to pool the data with sensitive analysis or subgroup analysis as we mentioned below. Even, we may ignore the heterogeneity during meta-analysis with reasonable explanation. If meta-analysis is not available due to the very significant heterogeneity among studies which we cannot afford to ignore, forest plot will still be shown without the pooling step, which may help readers to get an overview of the size and effects seen in the different studies. And based on the results from each single study, we plan to qualitatively describe the synthesis of the results. Whether the meta-analysis failed to be carried out or be modified according to the heterogeneity, we will discuss the limitations and the potential impact on the final results.

Subgroup meta-analysis will be conducted by factors that may contribute to the differences in the results, including study design (retrospective or prospective), characteristics of participants (gender, age, history of infertility), dose of caffeine intake, if data are available. Dose-response meta-analysis will be conducted if data permit. Sensitive analyses will be conducted to determine whether the conclusions are robust to arbitrary decisions made regarding the methodological quality (according to the ROB and NOS assessment) or the publishing time of the studies (within 5 years or not). Publication bias will be detected through funnel plot when included trials are more than ten [31].

### Overall quality of body of evidence: summary of findings table

Summary of findings (SOF) tables will be generated using the Guideline Development Tool (GDT) online program. The SOF table evaluates the overall quality of the body of evidence for clinical outcomes only from the results of the meta-analysis, which will use the Grading of Recommendations, Assessment, Development and Evaluation (GRADE) criteria [32]. The level of the evidence from randomized (or non-randomized) controlled trials would be downgraded based on study quality limitations, inconsistency of effect, imprecision, indirectness, and potential publication bias, and the level of the evidence from observational studies (including cohort study and case-control study) would be upgraded if there are large effects of the intervention/exposure according to the pooling results or potential uncontrolled confounding bias may weaken the true effect of the intervention/exposure. The GRADE is designed to rate the quality of a body of evidence and can be applied to evaluate systematic reviews and other forms of evidence.

## Discussion

### Key points of the review

This is a protocol of a systematic review of both observational studies and interventional studies. Different from systematic review of only randomized controlled trials, the methods of this review would be adopted according to the characteristics of observational studies. Besides the difference of methods on screening literatures, assessing the quality of included studies, analyzing the data, and reporting the results, the most important thing for observational studies is to control the confounders among exposure factors and risk. As a complicated condition, infertility may be caused by plenty of reasons (including the lifestyle and diet), and caffeine intake could be one of them. Thus, all the other factors beside caffeine intake should be measured and reported in the original included studies. Whether or not they analyzed the data regarding the impact of those factors, we will include those which meet our inclusion criteria but only pool the data adjusted for pre-specified confounders.

### Quality control of the final review

The report of the final review will be in accordance with both the Meta-Analysis of Observational Studies in Epidemiology (MOOSE) [33] and the Preferred Reporting Items for Systematic Reviews and Meta-Analyses (PRISMA) checklists statement [34].

A measurement tool to assess systematic reviews (AMSTAR) statement [35] will be employed to guide the methodology when conducting the review to avoid potential bias.

## Additional files

Additional file 1: Table S1. Data extraction form for randomized/quasi-randomized controlled trials or non-randomized clinical studies. (DOC 72 kb) Additional file 2: Table S2. Data extraction form for case-control study. (DOC 99 kb) Additional file 3: Table S3. Data extraction form for cohort study. (DOC 101 kb)

## Abbreviations

AMSTAR: a measurement tool to assess systematic reviews
CADTH: Canadian Agency for Drugs and Technologies for Health
CBM: Chinese Biomedical Database
CI: confidence interval
CNKI: China National Knowledge Infrastructure
GDT: Guideline Development Tool
GRADE: Grading of Recommendations, Assessment, Development and Evaluation
MeSH: Medical Subject Headings
MOOSE: Meta-Analysis of Observational Studies in Epidemiology
NOS: Newcastle-Ottawa Scale
PRISMA: Preferred Reporting Items for Systematic Reviews and Meta-Analyses
RCT: randomized controlled trial
ROB: risk of bias
RR: relative risk
SOF: summary of findings

## References

[1] “WHO|Infertility”. 2013. Accessed on 24 May 2015. http://www.who.int/reproductivehealth/topics/infertility/definitions/en/

[2] Mascarenhas MN, Flaxman SR, Boerma T, Vanderpoel S, Stevens GA (2012). National, regional, and global trends in infertility prevalence since 1990: a systematic analysis of 277 health surveys. PLoS Med.

[3] Maheshwari A, Hamilton M, Bhattacharya S (2008). Effect of female age on the diagnostic categories of infertility. Hum Reprod.

[4] Makar RS, Toth TL (2002). The evaluation of infertility. Am J Clin Pathol.

[5] Sunderam S, Kissin DM, Flowers L, Anderson JE, Folger SG (2012). Assisted reproductive technology surveillance—United States, 2009. MMWR.

[6] Kulkarni G, Mohanty NC, Mohanty IR, Jadhav P, Boricha BG (2014). Survey of reasons for discontinuation from in vitro fertilization treatment among couples attending infertility clinic. J Hum Reprod Sci.

[7] Fatima P, Rahman D, Hossain HB, Hossain HN, Mughi CR (2015). Psychosocial consequences of infertility on infertile women. Mymensingh Med J.

[8] Greenwood DC, Thatcher NJ, Ye J, Garrard L, Keogh G, King LG (2014). Caffeine intake during pregnancy and adverse birth outcomes: a systematic review and dose-response meta-analysis. European J Epidemiol.

[9] Cano-Marquina A, Tarín JJ, Cano A (2013). The impact of coffee on health. Maturitas.

[10] Qi H, Li S (2014). Dose-response meta-analysis on coffee, tea and caffeine consumption with risk of Parkinson's disease. Geriatrics Gerontology Int.

[11] Bolúmar F, Olsen J, Rebagliato M, Bisanti L (1997). Caffeine intake and delayed conception: a European multicenter study on infertility and subfecundity. European Study Group on Infertility Subfecundity. Am J Epidemiol.

[12] Chavarro JE, Rich-Edwards JW, Rosner BA, Willett WC (2009). Caffeinated and alcoholic beverage intake in relation to ovulatory disorder infertility. Epidemiology.

[13] Kinney A, Kline J, Kelly A, Reuss ML, Levin B (2007). Smoking, alcohol and caffeine in relation to ovarian age during the reproductive years. Hum Reprod.

[14] Stanton CK, Gray RH (1995). Effects of caffeine consumption on delayed conception. Am J Epidemiol.

[15] Sharma R, Biedenharn KR, Fedor JM, Agarwal A (2013). Lifestyle factors and reproductive health: taking control of your fertility. Reprod Biol Endocrinol.

[16] Buck GM, Sever LE, Batt RE, Mendola P (1997). Life-style factors and female infertility. Epidemiology.

[17] Anderson K, Nisenblat V, Norman R (2010). Lifestyle factors in people seeking infertility treatment—a review. Aust N Z J Obstet Gynaecol.

[18] Wilcox A, Weinberg C, Baird D (1988). Caffeinated beverages and decreased fertility. Lancet.

[19] Dorostghoal M, Erfani Majd N, Nooraei P (2012). Maternal caffeine consumption offspring rats. Clin Exp Reprod Med.

[20] Vacca G (1926). Riserche sulla alterazioni testicolari nell' awelanameuto speri mentale da caffeina. Arch Farmacol Sper Sci Affin.

[21] Stieve H (1931). Untersuchungen ueber die wechselbeziehungen zwischen gesamt korper und keimdrusen. Akad Verl Ges.

[22] Ezzat AR, el-Gohary ZM (1994). Hormonal and histological effects of chronic caffeine administration on the pituitary-gonadal and pituitary-adrenocortical axes in male rabbits. Funct Dev Morphol.

[23] Parazzini F, Marchini M, Tozzi L, Mezzopane R, Fedele L (1993). Risk factors for unexplained dyspermia in infertile men: a case-control study. Arch Androl.

[24] Fulgoni VL, Keast DR, Lieberman HR (2015). Trends in intake and sources of caffeine in the diets of US adults: 2001–2010. Am J Clin Nutr.

[25] Rudolph E, Faerbinger A, Koenig J (2014). Caffeine intake from all sources in adolescents and young adults in Austria. Eur J Clin Nutr.

[26] Wells GA, Shea B, O’Connell D, et al. The Newcastle-Ottawa Scale (NOS) for assessing the quality if nonrandomized studies in meta-analysis. Available at: http://www.ohri.ca/programs/clinical_epidemiology/oxford.htm. [Accessed December 20 2015].

[27] Higgins JPT, Green S. Cochrane handbook for systematic reviews of interventions version 5.0.2. The Cochrane collaboration. Available at: http://www.cochrane-handbook.org. [Accessed December 20 2015].

[28] Ioannidis JP, Patsopoulos NA, Evangelou E (2007). Uncertainty in heterogeneity estimates in meta-analyses. BMJ.

[29] Mittlböck M, Heinzl H (2006). A simulation study comparing properties of heterogeneity measures in meta-analyses. Stat Med.

[30] Brockwell SE, Gordon IR (2001). A comparison of statistical methods for meta-analysis. Stat Med.

[31] Sterne JA, Gavaghan D, Egger M (2000). Publication and related bias in meta-analysis: power of statistical tests and prevalence in the literature. J Clin Epidemiol.

[32] Schünemann H, Brożek J, Guyatt G, Oxman A, editors. GRADE handbook for grading quality of evidence and strength of recommendations. The GRADE Working Group. http://www.guidelinedevelopment.org/handbook. [Accessed December 20 2015].

[33] Stroup DF, Berlin JA, Morton SC, Olkin I, Williamson GD, Rennie D, Moher D, Becker BJ, Sipe TA, Thacker SB (2000). Meta-analysis of observational studies in epidemiology: a proposal for reporting. JAMA.

[34] Moher D, Liberati A, Tetzlaff J, Altman DG, PRISMA Group (2009). Preferred reporting items for systematic reviews and meta-analyses: the PRISMA statement. PLoS Med.

[35] Shea BJ, Grimshaw JM, Wells GA, Boers M, Andersson N, Hamel C, Porter AC, Tugwell P, Moher D, Bouter LM (2007). Development of AMSTAR: a measurement tool to assess systematic reviews. BMC Medical Res Methodology.

