# Supplementary material for: Is caffeine intake a risk factor leading to infertility? A protocol of an epidemiological systematic review of controlled clinical studies
Source: Syst Rev. 2016 Mar 15;5:45. doi: 10.1186/s13643-016-0221-9 (PMC4791877; doi:10.1186/s13643-016-0221-9)
Supplement: Additional file 1: Table S1. — Data extraction form for randomized/quasi-randomized controlled trials or non-randomized clinical studies. (DOC 72 kb) [file 13643_2016_221_MOESM1_ESM.doc]

# Randomized/Non-Randomized Controlled Studies

# Data Extraction form for *Study ID*

Data extractor:

Date of data extraction:

1^st^ checker of data extraction:

2^nd^ checker of data extraction:

| **Complete reference** | | |  | | | | | | | | | | |  |
| --- | --- | --- | --- | --- | --- | --- | --- | --- | --- | --- | --- | --- | --- | --- |
| Publication type | | |  | | | | | | | | | | |  |
| Contact author | | |  | | | | | | | | | | |  |
| Contact details | | |  | | | | | | | | | | |  |
| Country of study | | |  | | | | | | | | | | |  |
| Citation | | |  | | | | | | | | | | |  |
| **1. Type of study** | |  | | | | | | | | | | | | |
| **2. Inclusion criteria** | |  | | | | | | | | | | | | |
| **3. Exclusion criteria** | | | |  | | | | | | | | | | |
| **4.** | **Was a sample size calculation carried out?** | | | | | | | | |  | | |  | |
| **5. Intervention** | |  | | | | | | | | | | | | |
|  | caffeine type（tea、coffee、coke） | | | | | | | | |  | | |  | |
|  | Dose（day、week、month） | | | | | | | | |  | | |  | |
|  | Method of intake | | | | | | | | |  | | |  | |
|  | Duration of caffeine intake | | | | | | | | |  | | |  | |
|  | Additional information | | | | | | | | |  | | |  | |
| **6. Comparison** | |  | | | | | | | | | | | | |
| **7. Outcome measures** | |  | | | | | | | | | | | | |
| **8. Diagnostic criteria for infertility** | | | | | | |  | | | | | | | |
| **9. Follow up duration** | |  | | | | | | | | | | | | |
| **10. Recruitment procedures** | | | | |  | | | | | | | | | |
| **11. Setting** | |  | | | | | | | | | | | | |
| **12. Funding source** | |  | | | | | | | | | | | | |
| **13. Notes** | |  | | | | | | | | | | | | |
| **14. Quality assessment** | | | | | | | | | | | | | | |
| Random allocation | | | | | |  | | | | | | | | |
| Concealed allocation | | | | | |  | | | | | | | | |
| Baseline comparability | | | | | |  | | | | | | | | |
| Blind subjects | | | | | |  | | | | | | | | |
| Blind therapists | | | | | |  | | | | | | | | |
| Blind assessors | | | | | |  | | | | | | | | |
| Adequate follow-up | | | | | |  | | | | | | | | |
| Intention-to-treat analysis | | | | | |  | | | | | | | | |
| Between-group comparisons | | | | | |  | | | | | | | | |
| Point estimates and variability | | | | | |  | | | | | | | | |
| **15. Baseline characteristics** | | | | | | | | | | | | | | |
|  | | | | | | **Intervention**  **(N= )** | | | | | Comparison **(N= )** | | | |
| Age | | | | | |  | | | | |  | | | |
| Sex | | | | | |  | | | | |  | | | |
| How many child has been had | | | | | |  | | | | |  | | | |
| History of infertility in family | | | | | |  | | | | |  | | | |
| Time of marriage | | | | | |  | | | | |  | | | |
| Other potential confounders control | | | | | |  | | | | |  | | | |
| **16. Pre-treatment group differences** | | | | | | | |  | | | | | | |
| **17. Results** | | | | | | | | | | | | | | |
|  | | | | | | **Intervention** | | | | | Comparison | | | |
| Total randomised | | | | | |  | | | | |  | | | |
| Lost to follow up/exclusion* | | | | | |  | | | | |  | | | |
| Included in analysis | | | | | |  | | | | |  | | | |
| **Results for infertility** | | | | | | | | | | | | |  | |
|  | | | **Intervention 1**  **(N= )** | | | | | | **Intervention 2**  **(N= )** | | | Comparison **(N= )** |  | |
|  | | |  | | | | | |  | | |  |  | |
|  | | |  | | | | | |  | | |  |  | |
| Results for relative risk among groups | | | | | | | | | | | | |  | |
| Comparison 1 | | |  | | | | | |  | | |  |  | |
| Comparison 2 | | |  | | | | | |  | | |  |  | |
| Results for relative risk among groups (Intention-to treat analysis) | | | | | | | | | | | | |  | |
| Comparison 1 | | |  | | | | | |  | | |  |  | |
| Comparison 2 | | |  | | | | | |  | | |  |  | |
