# Supplementary figures and images for: Is caffeine intake a risk factor leading to infertility? A protocol of an epidemiological systematic review of controlled clinical studies
Source: Syst Rev. 2016 Mar 15;5:45. doi: 10.1186/s13643-016-0221-9 (PMC4791877; doi:10.1186/s13643-016-0221-9)

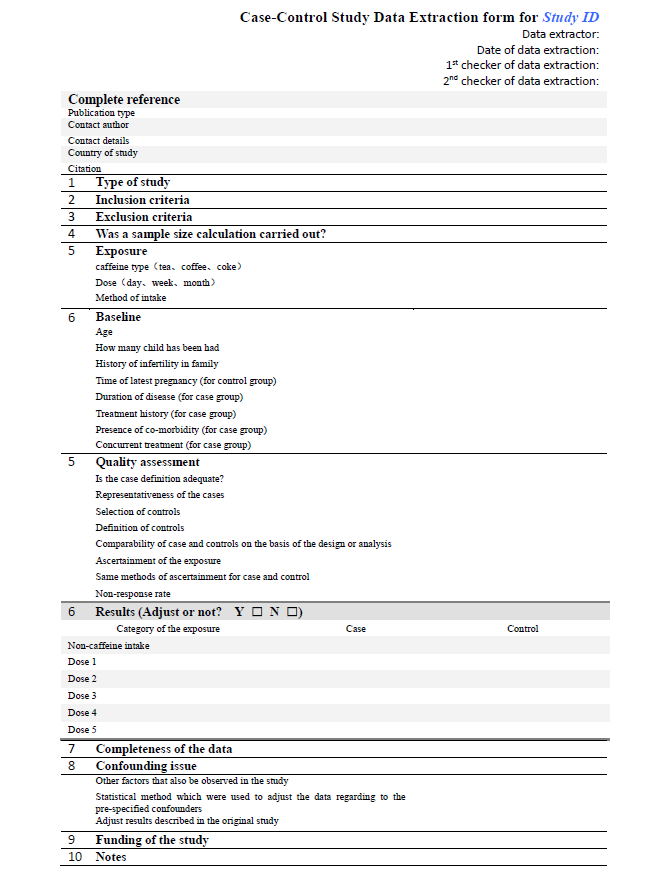

Supplement: Additional file 2: Table S2. — Data extraction form for case-control study. (DOC 99 kb) [file 13643_2016_221_MOESM2_ESM.doc]

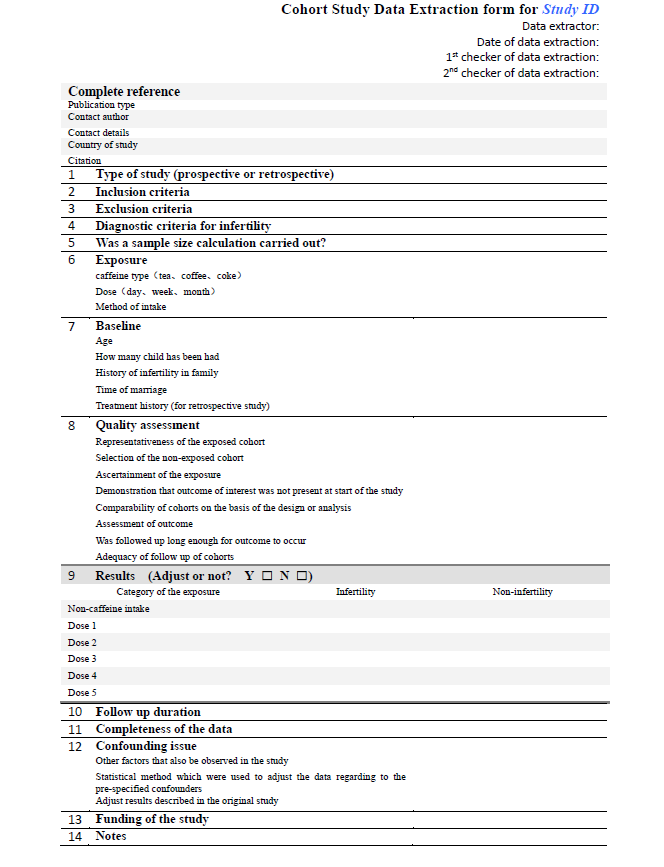

Supplement: Additional file 3: Table S3. — Data extraction form for cohort study. (DOC 101 kb) [file 13643_2016_221_MOESM3_ESM.doc]
